# Applying Classification Trees to Hospital Administrative Data to Identify Patients with Lower Gastrointestinal Bleeding

**DOI:** 10.1371/journal.pone.0138987

**Published:** 2015-09-25

**Authors:** Juned Siddique, Gregory W. Ruhnke, Andrea Flores, Micah T. Prochaska, Elizabeth Paesch, David O. Meltzer, Chad T. Whelan

**Affiliations:** 1 Department of Preventive Medicine, Northwestern University Feinberg School of Medicine, Chicago, Illinois, United States of America; 2 Department of Medicine, University of Chicago, Chicago, Illinois, United States of America; 3 Department of Medicine, Loyola University Stritch School of Medicine, Maywood, Illinois, United States of America; University of Catania, ITALY

## Abstract

**Background:**

Lower gastrointestinal bleeding (LGIB) is a common cause of acute hospitalization. Currently, there is no accepted standard for identifying patients with LGIB in hospital administrative data. The objective of this study was to develop and validate a set of classification algorithms that use hospital administrative data to identify LGIB.

**Methods:**

Our sample consists of patients admitted between July 1, 2001 and June 30, 2003 (derivation cohort) and July 1, 2003 and June 30, 2005 (validation cohort) to the general medicine inpatient service of the University of Chicago Hospital, a large urban academic medical center. Confirmed cases of LGIB in both cohorts were determined by reviewing the charts of those patients who had at least 1 of 36 principal or secondary International Classification of Diseases, Ninth revision, Clinical Modification (ICD-9-CM) diagnosis codes associated with LGIB. Classification trees were used on the data of the derivation cohort to develop a set of decision rules for identifying patients with LGIB. These rules were then applied to the validation cohort to assess their performance.

**Results:**

Three classification algorithms were identified and validated: a high specificity rule with 80.1% sensitivity and 95.8% specificity, a rule that balances sensitivity and specificity (87.8% sensitivity, 90.9% specificity), and a high sensitivity rule with 100% sensitivity and 91.0% specificity.

**Conclusion:**

These classification algorithms can be used in future studies to evaluate resource utilization and assess outcomes associated with LGIB without the use of chart review.

## Introduction

Lower gastrointestinal bleeding (LGIB) is a common cause of acute hospitalization with estimated annual incidence rates of 21 per 100,000 adults per year [1]. LGIB bleeding involves bleeding from the lower intestines to the anus and can be caused by hemorrhoids, cancer, polyps, and colitis, among other causes. LGIB occurs primarily in elderly patients and can result in substantial morbidity and mortality [2]. There are a limited number of studies that investigate the etiology of LGIB hospitalization, and its costs, outcomes and resource utilization are not well understood [3]. While administrative databases have historically been used by researchers and payers to answer these types of questions, health systems are increasingly interested in using these data sources to measure quality, cost, and efficiency as well.

Hospital administrative databases are an easily available and valuable data source for research on the epidemiology of various medical conditions such as LGIB. These databases often include multiple fields specifying principal and secondary diagnoses as well as principal and secondary procedures performed during the hospitalization. Diagnoses and procedures are typically coded using the *International Classification of Diseases, Ninth revision, Clinical Modification* (ICD-9-CM). Administrative databases also include some patient characteristics.

A challenge in using administrative databases for the analysis of LGIB hospitalization is that there is no accepted standard for identifying LGIB patients either through ICD-9-CM codes or Current Procedural Terminology (CPT) codes [4]. Prior studies that have most accurately identified patients with LGIB use a 2-step method to address this issue. The initial step aims to capture all potential LGIB cases by specifying a large number of ICD-9-CM codes that are associated with LGIB. The patients that meet this initial criteria then undergo chart review to confirm the presence of LGIB [1, 3, 5]. This method is expensive and time consuming and cannot be done using administrative databases alone.

The objective of this study is to develop and validate a set of classification algorithms for the identification of LGIB using hospital administrative databases. We began by identifying patients with LGIB using a 2-step method with both administrative data and chart review from two years of admissions data from a large urban academic medical center. We then used machine learning methods to derive a set of decision rules for classifying LGIB patients. These decision rules only require administrative data. We validated our decision rules using a new set of data from the subsequent two years of admissions at the same hospital. Our goal is to provide researchers with the ability to accurately identify LGIB cases in hospital administrative data without having to use chart review.

## Materials and Methods

### Participants and study design

We used data from the University of Chicago Hospitalist Project, an ongoing study of issues related to hospital care [6]. Our sample is drawn from those patients who were admitted between July 1, 2001 and June 30, 2003 (derivation cohort) and those patients admitted between July 1, 2003 and June 30, 2005 (validation cohort) to the general medicine inpatient service of the University of Chicago Hospital, a large urban academic medical center. The administrative data at the University of Chicago Hospital includes variables for age, gender, inpatient mortality, and contains one principal diagnosis field and 30 secondary diagnosis fields. There is also one principal procedure field and 30 secondary procedure fields. Both diagnosis and procedure fields use codes from the ICD-9-CM. Only a principal diagnosis is required for every patient. Patients who had missing administrative data, missing chart data, or were transfers from another hospital were excluded. Transfer patients were excluded since their procedures were performed at the transferring hospital and would not be captured in our database.

### Identifying LGIB using chart review

We used chart review as our gold standard for identifying patients with LGIB. To do this, all patients were first screened for a principal or secondary diagnosis of LGIB based on the 36 ICD-9-CM codes listed in Table 1. These codes were selected for a very high sensitivity threshold to assure that all potential subjects with LGIB were identified. If a patient received one of these target ICD-9-CM codes, they then underwent a detailed chart abstraction to confirm the presence of LGIB.

**Table 1:** ICD-9-CM codes used to screen patients with potential lower gastrointestinal bleeding. The third and fourth columns use data from the derivation cohort (n = 6,974).

Code	Description	No. with Principal Dx Secondary Dx (% w/LGIB)	No. with Secondary Dx (% w/LGIB)
003.0	Salmonella gastroenteritis	0 (NA)	1 (0%)
006.2	Amebic nondysenteric colitis	0 (NA)	0 (NA)
153.0	Hepatic flexure	0 (NA)	1 (0%)
153.1	Malignant neoplasm transverse colon	2 (0%)	0 (NA)
153.3	Malignant neoplasm sigmoid colon	2 (0%)	1 (0%)
153.4	Malignant neoplasm cecum	3 (100%)	0 (NA)
153.8	Malignant neoplasm colon, other specified site	1 (100%)	1 (0%)
153.9	Malignant neoplasm colon, unspecified	1 (100%)	7 (14%)
211.3	Benign neoplasm of colon	12 (25%)	103 (46%)
455.0	Internal hemorrhoid without complication	1 (100%)	38 (66%)
455.2	Internal hemorrhoids with other complication	7 (100%)	11 (82%)
455.5	External hemorrhoids with other complication	3 (100%)	8 (50%)
455.8	Unspecified hemorrhoids with other complication	2 (100%)	8 (88%)
456.8	Varices of other sites	1 (0%)	4 (0%)
556.2	Ulcerative proctitis	1 (0%)	1 (0%)
556.3	Ulcerative proctosigmoiditis	1 (100%)	0 (NA)
556.5	Left-sided ulcerative colitis	0 (NA)	1 (0%)
556.6	Universal ulcerative colitis	2 (50%)	4 (0%)
556.8	Other ulcerative colitis	2 (100%)	3 (67%)
556.9	Ulcerative colitis, unspecified	12 (67%)	12 (25%)
558.1	Radiation gastroenteritis	5 (40%)	10 (0%)
558.2	Toxic gastroenteritis	0 (NA)	0 (NA)
558.9	Other noninfectious gastroenteritis	29 (17%)	62 (13%)
562.02	Diverticulosis of small intestine with hemorrhage	2 (50%)	2 (100%)
562.03	Diverticulitis of small intestine with hemorrhage	0 (NA)	0 (NA)
562.10	Diverticulosis of colon without hemorrhage	4 (25%)	181 (26%)
562.12	Diverticulosis of colon with hemorrhage	73 (97%)	14 (71%)
562.13	Diverticulitis of colon with hemorrhage	8 (88%)	1 (0%)
569.3	Hemorrhage of rectum and anus	3 (100%)	16 (38%)
569.82	Ulceration of intestine	1 (100%)	5 (80%)
569.85	Angiodysplasia of intestine with hemorrhage	12 (83%)	8 (13%)
569.89	Other specified intestinal disorders	0 (NA)	15 (27%)
569.9	Unspecified disorder of intestine	0 (NA)	7 (29%)
578.1	Blood in stool	60 (67%)	160 (41%)
578.9	Gastrointestinal hemorrhage, unspecified	41 (59%)	41 (27%)
751.0	Meckel’s diverticulum	0 (NA)	0 (NA)

Dx = diagnosis; LGIB = lower gastrointestinal bleeding; NA = not applicable

Trained research assistants and clinicians performed the chart abstractions with validation by senior clinicians (C.T.W and G.W.R) of the first 15 charts to ensure accuracy. Subsequently, abstractors consulted with senior clinicians regarding any questions during abstracting with final decisions being made by the senior clinicians. Criteria for chart-validated LGIB included (i) the patient presented with black, maroon or bloody stool and had a diagnostic study whose result was consistent with LGIB; or (ii) the medical team documented LGIB or probable LGIB in the discharge summary.

### Identifying LGIB using classification trees

Our derivation cohort consists of 6,974 patients admitted between 2001 and 2003. Among these patients, there were 1078 unique principal diagnoses, 2,661 unique secondary diagnoses, 378 unique principal procedures, and 508 unique secondary procedures. Without some form of regularization [7], traditional parametric classification methods such as logistic regression or discriminant analysis would fail in a model with 6,974 observations and 4,628 covariates. Considering interactions among these covariates is even more difficult [8]. Therefore, we chose to use classification trees [9] to develop our decision rules.

Classification trees are a computationally intensive nonparametric approach to classification often used in medical decision-making. A key advantage of classification trees as compared to other machine learning methods is that they are fast to construct and provide interpretable models [10]. Tree construction consists of searching through covariates and choosing the one variable that best subsets the data into two groups (nodes) in which the distribution of the variable to be classified is more *pure* (see definition of purity below) in each node than before the split. If a covariate is not already binary, the algorithm identifies the cutoff that provides the best split. Once the data have been subset into two nodes, the algorithm then considers each node separately. If a node is sufficiently pure, no more classification is necessary. Otherwise, the algorithm—using only the cases in that node—searches through the remaining variables to identify the next variable that best splits the node into two additional nodes of increased purity. This construction process continues until all nodes are sufficiently pure and no more splitting is necessary. Nodes that no longer need to be subset are called terminal nodes. Each terminal node is thus identified by a series of binary splits of variables in the data set and the resulting classification is a set of decision rules that are easy to understand and implement.

The decision to continue splitting a parent node into two child nodes is based on whether splitting the parent node results in a decrease in impurity. Let *i*(*t*) be the impurity function in node t and define Δ*i*(*s*,*t*) as the change in impurity resulting from using variable *s* to split node *t* into two child nodes *t*_*L*_ and *t*_*R*_. Then the classification tree algorithm maximizes the change in impurity:

$$\Delta i\left(s,t\right)=i\left(t\right)-p_{L}i\left(t_{L}\right)-p_{R}i\left(t_{R}\right)$$

where *p*_*L*_ and *p*_*R*_ are the proportion of cases from node *t* that went into nodes *t*_*L*_ and *t*_*R*_ respectively.

There are several choices for the impurity function. We used the Gini Index as recommended by Breiman et al. [9]. In our case, where there are only two classes (LGIB, non-LGIB), the impurity of a node using the Gini Index is simply *p*(1−*p*) where *p* is the proportion of cases in one of the classes.

When dealing with low-rate events in data such as ours, where the prevalence of LGIB is 4% among all candidate admissions, a tree that gives equal importance to correctly classifying both LGIB and non-LGIB patients will result in a tree with very high specificity, but low sensitivity. To see this, assume we have a data set with 4 patients with LGIB and 96 without LGIB. A tree that misclassifies 1 LGIB patient and 1 non-LGIB patient will result in a tree with an overall misclassification rate of 2/100 = 2%, but 25% of the LGIB patients will be misclassified (75% sensitivity) and only 1/96 of the non-LGIB patients will be misclassified (99% specificity).

In order to build trees with higher sensitivity (at the expense of specificity), one approach is to give more weight to the LGIB observations and less weight to the non-LGIB observations. Weighting has the effect of altering the impurity function *i*(*t*) so that LGIB observations contribute more to node impurity than non-LGIB observations. The result is that the tree is grown so that splits are chosen that favor correct classification of LGIB at the expense of non-LGIB [9]. Giving more weight to LGIB observations is equivalent to increasing the cost of misclassifying an LGIB case as a non-LGIB case. For example, giving LGIB observations twice as much weight as non-LGIB observations is equivalent to specifying that the cost of misclassifying an LGIB case as a non-LGIB case is twice that of misclassifying a non-LGIB case as a LGIB case [11]. As the cost of misclassifying an LGIB case as a non-LGIB case increases, so does the sensitivity of the resulting tree (at the expense of the tree’s specificity). In this way we are able grow trees with different sensitivities and specificities.

A useful summary of a weighting function is the weighted proportion of LGIB patients in our sample, referred to as the prior probability of LGIB. When all observations are given equal weight, the prior probability of LGIB is simply the proportion of LGIB cases (4% in our sample). Giving more weight to LGIB cases and less weight to non-cases (i.e. increasing the relative cost of misclassifying an LGIB case as a non-LGIB case) will increase this prior probability. By using different prior probabilities (priors), we can control the tradeoff between sensitivity and specificity to obtain decision rules that either maximize sensitivity, maximize specificity, or provide a balance between the two. Classification trees were fit using the ‘rpart’ package [12] in the software program R [13]. Note that the classification trees had access to all the diagnosis and procedure codes in the data, not just the 36 diagnosis codes in Table 1.

Based on the use of different priors, we constructed a receiver operating characteristic (ROC) curve by plotting sensitivity against the false positive rate (1− specificity) over the range of priors in order to visualize the sensitivity/specificity tradeoff. Using the ROC curve, we chose two decision rules resulting from two classification trees: 1) a tree that maximizes specificity; 2) a tree that balances both sensitivity and specificity. We did not grow a tree that maximizes sensitivity because we found that using all the ICD-9-CM codes in Table 1 provided 100% sensitivity (by design, since these were the only patients whose charts were abstracted) with good specificity.

### Validation of classification rules

An important issue in tree construction is determining at which point to stop growing the tree. A very large tree might overfit the data, providing a classification rule that does not perform well on a new set of data. A too small tree might not do as good a job classifying observations as a tree that uses more classification variables. We set the minimum terminal node size to 7 observations and performed cost-complexity pruning with tenfold cross-validations to find the right-sized tree. See Breiman et al. [9] for details.

Although cost-complexity pruning helps to avoid growing a tree that overfits the data, a better measure of the utility of a decision rule is its external validity: how well it performs on data that was not used to develop the classification tree. Therefore, we performed another set of chart abstractions on the patients admitted between July 2003 and June 2005 who met our inclusion criteria. We then applied the classification rules that were developed on the 2001–2003 cohort to the 2003–2005 cohort to see if the rules performed well on an external sample. These rules were applied directly to the validation cohort without modification. Since the decision rules were developed only using the derivation data, the decision rules do not use any diagnoses codes that are in the validation cohort but not the derivation cohort.

This study was approved by the University of Chicago Institutional Review Board. Participants or their proxies provided written consent.

## Results

**Fig 1:**
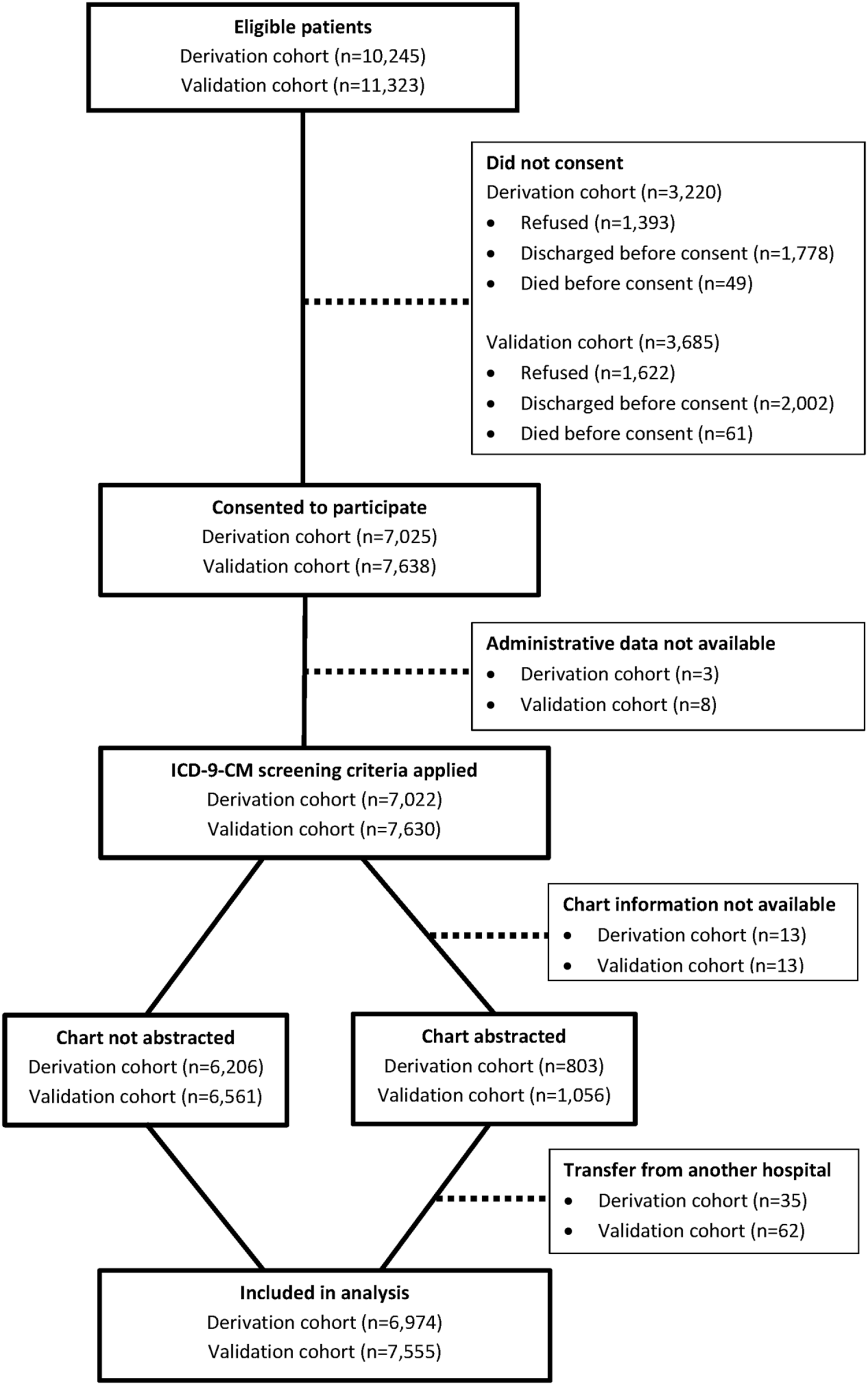
Participant flow diagram. Derivation cohort (July 2001—June 2003) and validation cohort (July 2003—June 2005).

Fig 1 is a flow diagram depicting the screening and enrollment process of our study. Between July 2001 and June 2003, 10,245 patients were admitted to the general medicine inpatient service at the University of Chicago Hospital. Of these, 3,220 were not consented, mostly because they refused consent or because they were discharged before consent could be obtained. Eventually, 6,974 patients were included in our derivation cohort. The numbers in the validation cohort were similar, resulting in 7,555 patients in this sample.

Table 2 describes the patient characteristics of our derivation and validation cohorts. The average age in both cohorts was 58 years. Patients were mostly female, and inpatient mortality was low (1.3%). An average patient in our derivation data set received 8.0 secondary diagnoses and 73% of patients received a principal procedure code. The average number of secondary procedures was 1.1. Approximately 12% in each cohort met the initial screening criteria based on having one of the 36 ICD-9-CM codes in Table 1 as a principal or secondary diagnosis. Eventually, 4.4% of our sample was determined to have LGIB.

**Table 2:** Patient characteristics from derivation and validation cohorts. Patients from the derivation cohort were admitted between July 1, 2001 and June 30, 2005. Patients from the validation cohort were admitted between July 1, 2003 and June 30, 2005.

Variable	Derivation Cohort (n = 6,974)	Validation Cohort (n = 7,555)
Age, mean (SD)	58.0 (20.0)	58.2 (19.5)
Male, No. (%)	2,555 (36.6)	2,845 (37.7)
Inpatient mortality, No. (%)	93 (1.3)	98 (1.3)
Secondary diagnoses, mean (SD)	8.0 (4.6)	10.6 (3.7)
Principal procedure, No. (%)	5,068 (72.7)	5,142 (68.1)
Secondary procedures, mean (SD)	1.2 (1.9)	1.1 (2.0)
Met initial screening criteria, No. (%)	768 (11.0)	994 (13.2)
Confirmed LGIB, No. (%)	297 (4.3)	343 (4.5)

LGIB = lower gastrointestinal bleeding

The second and third columns of Table 1 show the number of patients in our derivation cohort with the principal and secondary diagnoses that were used in our initial screening criteria. The percentage in parenthesis is the proportion of these patients who had a confirmed case of LGIB. The most common codes were 562.12 (diverticulitis of colon with hemorrhage) and 578.1 (blood in stool). As we will see below, a principal diagnosis of 562.12 is an ideal classification variable as a large number of patients in our sample have this principal diagnosis and 97% of them have LGIB.

### Classification algorithms

**Fig 2:**
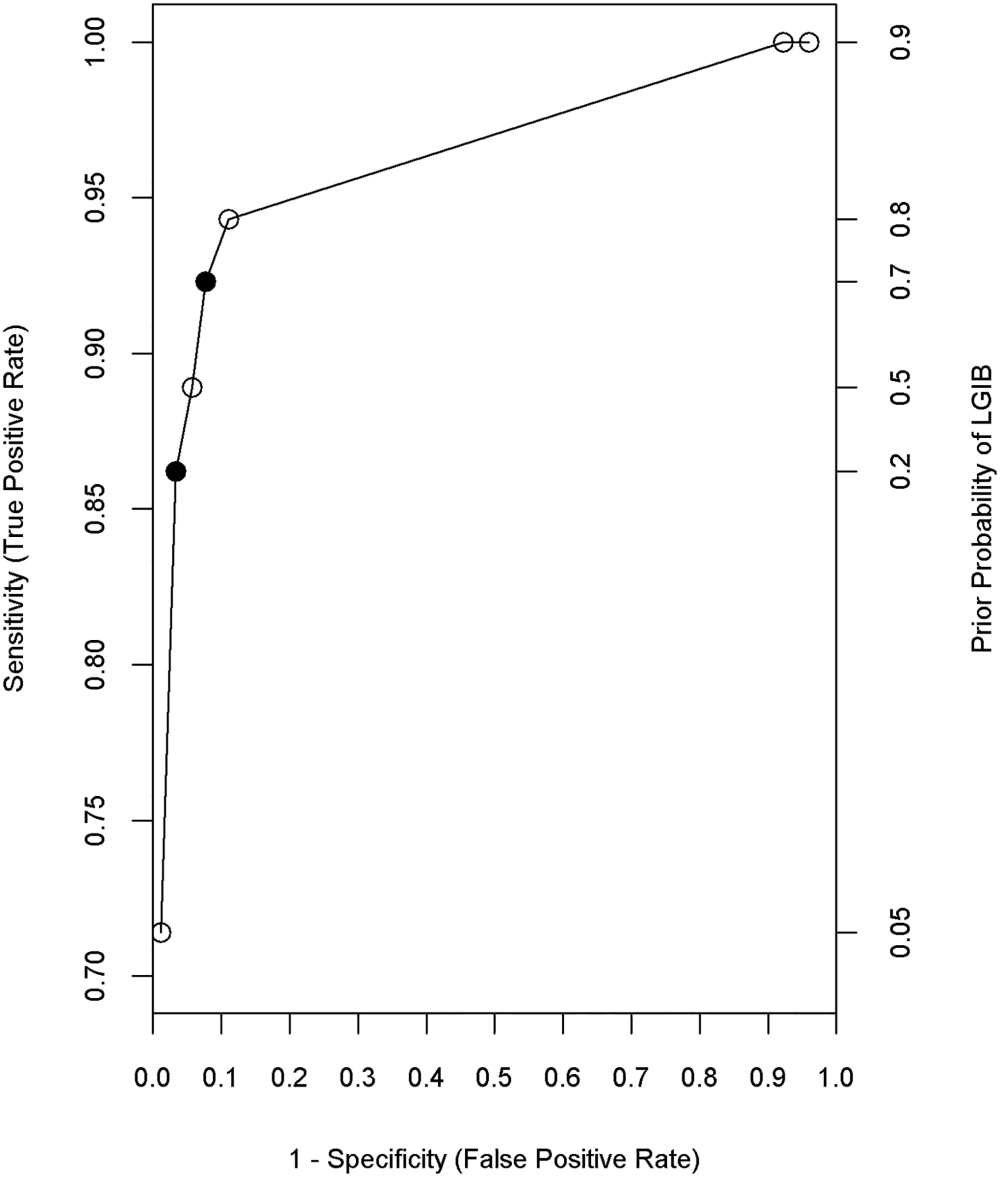
Receiver Operating Characteristic Curve. Each point along the curve represents a different classification tree generated by varying the prior probability of lower gastrointestinal bleeding (LGIB). The two solid points on the graph represent the trees that were selected to either maximize specificity (sensitivity = 86.2%, specificity = 96.7%) or to balance sensitivity and specificity (sensitivity = 92.3%, specificity = 92.3%).

Fig 2 is an ROC curve that presents sensitivity and specificity of decision rules based on specifying different prior probabilities for lower GI bleeding. Based on the results of these analyses, we chose two classification trees. The first tree was chosen because it provides high specificity with modest sensitivity. This tree was grown using a prior LGIB probability of 20% so that LGIB cases have 6 times the weight as non-LGIB cases (i.e. the cost of misclassifying a LGIB case is 6 times that of misclassifying a non-LGIB case) and results in a tree with 86.2% sensitivity and 96.7% specificity. The decision rules from this tree are presented in Table 3 and in S1 Fig in the Supporting Information. The decision rule depicted in Table 3 only requires 8 ICD-9-CM codes as compared to the 36 codes in Table 1.

**Table 3:** Results from a classification tree specifying a prior lower gastrointestinal bleeding probability of 20%. This tree provides 86.2% sensitivity and 96.7% specificity.

If/Else Statement	Principal Dx =	Secondary Dx =	Then Classify as	No. Correct	No. Incorrect
IF	562.12	–	LGIB	71	2
ELSE IF	–	578.1	LGIB	62	94
ELSE IF	578.1	–	LGIB	40	20
ELSE IF	578.9	–	LGIB	24	17
ELSE IF	569.85	–	LGIB	10	2
ELSE IF	–	562.12	LGIB	9	4
ELSE IF	–	211.3	LGIB	13	41
ELSE IF	–	578.9	LGIB	10	29
ELSE IF	–	455.2	LGIB	6	1
ELSE IF	556.9	–	LGIB	6	4
ELSE IF	–	455.0	LGIB	5	5
ELSE	–	–	Not LGIB	6458	41

Dx = diagnosis; LGIB = lower gastrointestinal bleeding

Looking at Table 3, we see that the classification tree identified principal diagnosis code 562.12 as the single best discriminator between LGIB and non-LGIB. Based on this single variable, 71 of 297 bleeds in our sample were correctly classified and only 2 patients were incorrectly classified as having LGIB. The next best predictor of LGIB is secondary diagnosis code 578.1. Among those patients who did not have a principal diagnosis of 562.12, 62 of the remaining 224 bleeds in our sample were correctly classified with LGIB and 94 were incorrectly classified with LGIB. As the tree moves downward, the remaining predictors are less discriminatory and fewer and fewer cases of LGIB are classified.

It is worth noting that a tree with one variable, principal diagnosis code 562.12, provides almost 100% specificity, but a sensitivity of 23.9%. We chose the tree described in Table 3 instead since it produced a substantial increase in sensitivity without much of a loss in specificity.

For ICD-9-CM codes 211.3, 455.0, and 455.2, only the secondary diagnosis is included in the tree, not the principal diagnosis. There are three possible reasons for this. First, these codes are relatively rare principal diagnoses. For example, only 1 participant among the 6,974 patients in our derivation cohort received a principal diagnosis code of 455.0. A terminal node this small is smaller than our minimum terminal node size and would likely be removed by cost-complexity pruning even if we set the minimum node size to one. Second, LGIB is more prevalent among patients who received these codes as secondary diagnoses as compared to principal diagnoses. For example, only 25% of the 12 patients who received code 211.3 as a principal diagnosis had LGIB while 46% of the 103 who received it as a secondary diagnosis had LGIB (Table 1). As a result, the secondary diagnosis is more useful than the principal diagnosis for classifying LGIB in our data. The final possibility is that patients with these principal diagnoses had already been classified by a secondary diagnosis code higher up in the decision tree. Four observations with the principal diagnosis code 211.3 and 2 observations with the principal diagnosis code 455.2 were classified based on a secondary diagnosis code higher up in the decision tree. All three reasons contribute to an ICD-9-CM code only appearing as a secondary diagnosis.

Table 4 is a classification tree based on a prior LGIB probability of 70% so that cases of LGIB have 52 times the weight as non-cases. This prior was chosen to balance both sensitivity and specificity. The sensitivity of the tree is 92.3% and the specificity is 92.3%. This tree consists of 8 unique ICD-9-CM diagnosis codes and 1 unique procedure code. The decision rules in Table 4 begin the same way as those in Table 3, but then change. The reason for this is that the tree in Table 4 is working harder to increase sensitivity. As a result, the tree chooses variables that identify a greater number of LGIBs, even at the expense of more false positives. Because our prior puts more weight on cases of LGIB, the tree algorithm still finds it worthwhile to continue expanding the tree, even if it is to only correctly classify 2 cases of LGIB.

**Table 4:** Results from a classification tree specifying a prior lower gastrointestinal bleeding probability of 70%. This tree provides 92.3% sensitivity and 92.3% specificity.

If/Else Statement	Principal Dx =	Secondary Dx =	Principal Proc =	Secondary Proc =	Then Classify as	No. Correct	No. InCorrect
IF	562.12	–	–	–	LGIB	71	2
ELSE IF	–	578.1	–	–	LGIB	62	94
ELSE IF	578.1	–	–	–	LGIB	40	20
ELSE IF	578.9	–	–	–	LGIB	24	17
ELSE IF	–	280.0	–	–	LGIB	22	136
ELSE IF	–	211.3	–	–	LGIB	12	39
ELSE IF	–	578.9	–	–	LGIB	9	24
ELSE IF	–	562.12	–	–	LGIB	8	3
ELSE IF	556.9	–	–	–	LGIB	6	4
ELSE IF	–	–	45.23	–	LGIB	6	22
ELSE IF	558.9	–	–	–	LGIB	4	22
ELSE IF	–	562.10	–	–	LGIB	5	96
ELSE IF	–	–	–	45.23	LGIB	3	29
ELSE IF	–	556.9	–	–	LGIB	2	9
ELSE	–	–	–	–	Not LGIB	6160	23

Dx = diagnosis; Proc = procedure; LGIB = lower gastrointestinal bleeding

As with the tree in Table 3, the tree in Table 4 contains ICD-9-CM codes where only the secondary diagnosis is included. Interestingly, one of these codes, 280.0 (Anemia secondary to chronic blood loss), was not one of the screening codes used in Table 1. Code 280.0 reflects a symptom of chronic LGIB (and other diseases) rather than a cause and was included in the tree despite the fact that it resulted in 136 cases misclassified as LGIB because LGIB cases in this tree are weighted so heavily. Table 4 also includes a procedure code, 45.23 (colonoscopy) in both the principal and secondary procedure fields.

Although Table 4 provides a parsimonious tree with good sensitivity and specificity, it does not outperform a decision rule that uses all the codes described in Table 1. Assigning LGIB to any patient who received at least one of the principal or secondary diagnosis in Table 1 results in a decision rule with 100% sensitivity and 92.9% specificity.

### Validation using Independent data

As discussed above, we performed another set of chart abstractions on the 7,555 patients who consented to the hospitalist study and were admitted to the University of Chicago Hospital between July 2003 and June 2005. The average number of secondary diagnoses for these patients is 10.6. Based on the chart abstractions, 343 of these patients were diagnosed with lower GI bleeding.

We applied the two classification rules (high specificity, balanced sensitivity and specificity) described in Tables 3 and 4 to the later data. Since the classification rules were developed based on data from July 2001 through June 2003, using the later data provides us with an external sample of data that we can use to validate our classification rules. The high specificity classification rule classified LGIB in the validation sample with 80.1% sensitivity and 95.8% specificity. The balanced classification rule classified lower GI bleeding in the validation cohort with 87.8% sensitivity and 90.9% specificity. Using all of the diagnosis codes in Table 1 classified LGIB with 100.0% sensitivity (by design) and 91.0% specificity.

## Discussion

The easy availability of hospital administrative databases offers the potential for studying a wide variety of diseases in a cost effective manner. The first step in using these databases is the identification of patients with the disease of interest. Using methods from machine learning, we demonstrated a practical approach for identifying patients with a given disease using hospital administrative data. By applying weights to our sample, we generated several different classification rules which provide a tradeoff between sensitivity and specificity. We validated our decision rules on a new data set, using administrative data from the subsequent two years of admissions. Our rules worked well on the validation data set, although not as well as on the data used to derive the rules.

This is the first study that we are aware of that has used machine learning methods to predict LGIB using administrative data. We anticipate our decision rules to be used for several different purposes. The high specificity rule in Table 3 will be useful when investigators wish to determine the prevalence of LGIB. Because sensitivity is low, classification based on this decision rule will miss some cases of LGIB. However, the high specificity will lower the number of false positives and provide a more accurate estimate of the prevalence of LGIB. This rule also performed well on our validation data set.

The classification rule in Table 4 balances both sensitivity and specificity and will miss fewer patients with LGIB than our high specificity rule. However, this rule provides lower specificity than the rule that uses all 36 ICD-9-CM codes in Table 1. Therefore, if the goal is to maximize sensitivity, then the rule based on Table 1 is the right approach. This approach would be useful when an investigator wishes to identify all possible cases of LGIB. Because specificity is not high (92.9%), prevalence estimates based on this classification rule will be biased upwards, especially in a large database. In the validation data set, using all the ICD-9-CM codes in Table 1 also provided higher sensitivity and specificity than the balanced classification rule.

LGIB is an amalgam of diagnoses and Table 1 highlights the difficulty of identifying LGIB in administrative databases. While a few codes make up about half of the cases of LGIB in our database, the other half are spread over a number of different codes representing a wide range of diseases. Further, for many people hospitalized with LGIB, the ICD-9-CM code related to LGIB is the secondary diagnosis rather than the principal diagnosis. As a result, developing an accurate and parsimonious decision rule for identifying LGIB is challenging. Still, the sensitivity and specificity of our rules were above 80% in all instances and compare favorably to other algorithms for determining case definition using administrative data in areas such as colonoscopy indication [14] and community acquired pneumonia [15], where identification of cases using ICD-9-CM codes is also problematic.

When plotted as a classification tree, the rules in Tables 3 and 4 consist of a single tree branch, and the decision rules are a series of IF/ELSE statements (or equivalently, OR statements). S1 Fig is a graphical display of the Table 3 decision rule. A primary reason for this structure is that a patient can only have one principal diagnosis so that a decision rule cannot consist of a combination of two principal diagnoses. However, the tree is not restricted to having a single branch and prior to beginning this project, we hypothesized that the decision rules would consist of AND statements such that a case would be defined by, for example, the presence of a principal diagnosis and a secondary diagnosis. After further exploration of our data, we realized that patients were admitted to the hospital for many different reasons and that our data are too sparse to support such a tree.

Specifically, the derivation cohort (n = 6,974) contains 1078 unique principal diagnosis ICD-9-CM codes and 2661 unique secondary diagnosis ICD-9-CM codes. The most common principal ICD-9-CM diagnosis, 428.0: Congestive heart failure, was diagnosed in only 4.8% of patients. Among the ICD-9-CM diagnosis codes in Table 1 which were used in our screening criteria, the most common principal diagnosis, 562.12: Diverticulosis of colon with hemorrhage, was diagnosed in only 1.0% of the derivation cohort. Secondary diagnoses were more common. The three most common secondary ICD-9-CM diagnoses from Table 1 were 562.10 (2.6% of the cohort); 578.1 (2.3% of the cohort); and 211.3 (1.5% of cohort) and this helps explain why some diagnosis codes only appear in our decision rules as secondary diagnoses—too few patients received these codes as principal diagnoses.

Cross-classifying principal and secondary diagnoses results in even more sparse data and does not necessarily result in more accurate classification. For example, the most common principal/secondary diagnosis combination in our data that used codes from Table 1 was principal = 578.1, secondary = 211.3 which was observed in 16 patients (0.23% of the sample). However the rate of LGIB among these participants was only 63%, lower than the rate (67%) among all patients with a principal diagnosis code of 578.1. Most other principal/secondary diagnoses combinations were rare, only appearing once—smaller than our minimum terminal node size.

Using classification trees to develop decision rules for identifying cases of LGIB has several advantages. First, classification trees are a non-parametric method that makes no assumptions about the underlying distributions of the predictors or the relationships between the predictors and the outcome. In addition, classification trees implicitly consider all possible interactions between potential predictors when building the algorithm and can easily handle situations where the number of covariates is greater than the number of observations. Finally, classification trees result in easy to communicate and use decision rules which provide for straightforward application in other settings.

There are numerous other machine learning methods that could have been used on our data to predict patients with LGIB [10]. One approach, boosting [16], is an extension of classification trees in which a weighted sum of many trees are used for classification rather than a single tree. In this way, boosting offers the potential for a smaller error rate as compared to using a single tree. However, the error rate of our high specificity tree is already quite small and a low error rate is not our only goal as evidenced by the fact that we were willing to accept increased error in exchange for higher sensitivity. In addition, a weighted sum of trees does not have the same interpretability of a single tree. We believe that interpretable and easily understood decision rules are necessary—in our setting—if they are to be applied by other researchers.

There are some important limitations to this study. Our results are from a single urban academic medical center with a patient population that is predominantly African American, which may limit the generalizability our findings. Our decision rules may work differently in other settings where the distribution of diagnoses and procedures is different. This study required consent and therefore only examines a subset of patients admitted to the medical center, which could potentially introduce bias into the sample. However, it is not clear why there would be systematic differences in subjects who choose to consent versus those who did not consent that would affect the results of this study in substantive ways.

As can be seen in Table 2, there is a significant difference in the average number of secondary diagnoses between the derivation (average of 8.0 secondary diagnoses) and validation cohorts (average of 10.6 secondary diagnoses). After further investigation, it appears that in 2003 there was an information systems upgrade at the University of Chicago Hospitals to make sure all secondary diagnoses were captured in the cost accounting systems. The result of this initiative was an increase in the number of secondary diagnoses. These types of upgrades in clinical and billing systems are common and in this sense, our validation cohort provides a “real-world” setting to validate our algorithm in which the validation cohort is unlikely to have a similar joint distribution of features and outcomes as the learning sample due to changes in the way administrative data are collected. A limitation is that the greater number of secondary diagnoses in the validation data may result in higher sensitivity and lower specificity of the decision rules as compared to having used a validation cohort with a similar distribution of secondary diagnoses as the derivation cohort.

While our initial screening criteria for chart review included all possible ICD-9-CM codes that we believed to be associated with LGIB, it is possible that there are patients in our sample with LGIB whose charts we did not abstract. While we believe this number to be small, our decision rules are unlikely to identify this group of patients. The rate of LGIB among those patients who did not receive one of the 36 ICD-9-CM screening codes in Table 1 is most likely substantially lower than the prevalence of 4.4% among the chart reviewed patients. Longstreth [1] estimated the general population hospitalization rate of LGIB as 20.5/100,000. We use 10 times this number (0.2%) as a possible upper bound for the rate of LGIB among those not chart reviewed. If we make the additional conservative assumptions that: 1) All of the additional 0.2% of cases are incorrectly classified by our algorithms and 2) the decrease in non-cases come from formally correctly-classified non-cases, then the sensitivity of our high-specificity rule would decrease from 86.2% to 82.3%. Under the same assumptions, sensitivity for our balanced sensitivity/specificity rule would decrease from 92.3% to 88.1%. The high sensitivity rule, rather than providing 100% sensitivity, would provide 95.5% sensitivity. For all three rules, specificity is essentially unchanged.

Although the specificity of our decision rules is high, they still resulted in a considerable number of falsely identified patients as having LGIB. With 7000 non-LGIB patients, each additional specificity percentage away from 100% results in 70 additional false positives. With a low prevalence disease like LGIB, these false positives can greatly effect estimates of prevalence. Specificity less than 100% also has a high impact on the positive predictive value of our decision rules. The high specificity decision rule in Table 3 has a positive predictive value of 53.9% and negative predictive value of 99.4%. For the decision rule in Table 4, these values are 34.6% and 99.6%, respectively. For the high sensitivity rule that uses all the ICD-9-CM codes in Table 1, the positive and negative predictive values are 38.7% and 100%, respectively.

Finally, our decision rules use the ICD-9-CM. In the United States, all programs covered by the Health Insurance Portability and Accountability Act (HIPAA) must transition to the ICD-10-CM coding system by October 1, 2015. It is unlikely that use of the ICD-10-CM will improve identification of LGIB and one study showed that the implementation of ICD-10 coding has not improved the quality of administrative data relative to the ICD-9-CM [17]. Still, our rules will only apply to the many years of data collected prior to the transition to the ICD-10-CM and future work will develop decision rules for identifying LGIB using the ICD-10-CM.

This study is particularly relevant in an era of increasing reliance on administrative data for policy decisions, quality measurement, and utilization assessment. Administrative data have the advantage of being readily available across multiple sites allowing for evaluations that are larger, faster, and cheaper than traditional chart review methods. Both public and private health care systems as well as payers are investing in methods to understand care patterns to assess quality, resource utilization, variability, and outcomes. To perform these analyses on this scale, using administrative data as the primary method is essential. Despite the significant advantages of administrative data, important limitations remain due to the fact that administrative data are typically not collected to address research questions. Methods that allow for a more nuanced use of these data are needed to minimize the impact of these limitations.

## Conclusions

We have shown that classification trees are a useful method of identifying patients with LGIB using administrative databases. These trees could be used by researchers, health systems, and policy makers to better understand the epidemiology of LGIB as well as treatment patterns, costs and clinical outcomes. The ability to generate trees with varying test characteristics allows users to choose the most appropriate sensitivity/specificity balance for their particular use. For example, using a balanced sensitivity/specificity tree might be most useful in examining if resources (such as endoscopy rooms) are appropriately distributed to match demand across a health system. If a specific hospital has high volumes of patients with LGIB compared with other system hospitals but has a smaller number of endoscopy rooms, it might be important to consider the need for investing in endoscopy resources, especially if rates of colonoscopies among patients with LGIB at that hospital are low or if length of stay is particularly high.

If the question is focused on individual physician practice patterns, a very high specificity tree would be a more appropriate method so that physician performance related to LGIB is measured only on patients who actually have LGIB. For example, if physicians were expected to assess coagulation parameters in all patients with LGIB, it would be essential to use a high specificity tree to identify target cases. Our decision rules have been tested and validated for LGIB and could be used directly for these purposes. Additionally, our approach could be used to identify other diagnoses and patient populations for research or outcomes assessment.

## Supporting Information

S1 Fig Classification tree for the high specificity decision rule. This tree is a graphical display of the high specificity rule shown in Table 3. The decision rule is a series of IF/ELSE statements such that the tree consists of a single branch.
(TIFF)
