# Supplementary figures and images for: Applying Classification Trees to Hospital Administrative Data to Identify Patients with Lower Gastrointestinal Bleeding
Source: PLoS One. 2015 Sep 25;10(9):e0138987. doi: 10.1371/journal.pone.0138987 (PMC4583289; doi:10.1371/journal.pone.0138987)

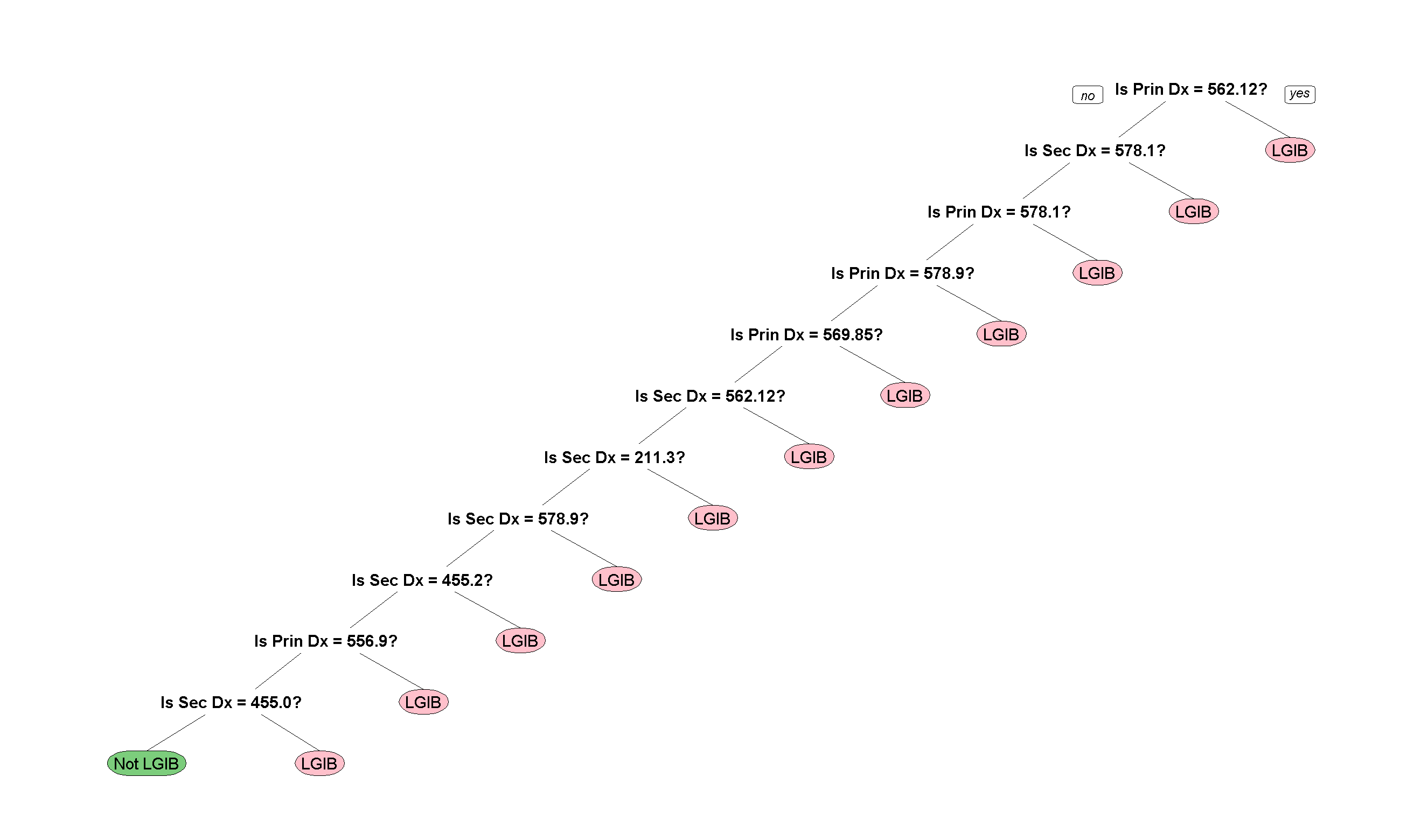

Supplement: S1 Fig — This tree is a graphical display of the high specificity rule shown in Table 3. The decision rule is a series of IF/ELSE statements such that the tree consists of a single branch. (TIFF) [file pone.0138987.s001.tiff]
